# Cancer risks associated with the germline MITF(E318K) variant

**DOI:** 10.1038/s41598-020-74237-z

**Published:** 2020-10-13

**Authors:** Samantha M. Guhan, Mykyta Artomov, Shelley McCormick, Ching -Ni Njauw, Alexander J. Stratigos, Kristen Shannon, Leif W. Ellisen, Hensin Tsao

**Affiliations:** 1 Wellman Center for Photomedicine at Massachusetts General Hospital, Edwards 211, 50 Blossom Street, Boston, MA 02114 USA; 2 MGH Analytic and Translational Genetics Unit, MGH and Broad Institute, Boston, MA USA; 3 Massachusetts General Hospital Cancer Center, Boston, MA USA; 4 First Department of Dermatology-Venereology, Faculty of Medicine, ‘A. Sygros’ Hospital for Cutaneous and Venereal Diseases, National and Kapodistrian University of Athens, Athens, Greece

**Keywords:** Cancer genetics, Skin cancer, Genetics

## Abstract

The MITF(E318K) variant confers moderate risk for cutaneous melanoma. While there are small studies suggesting that this risk is associated with other malignancies (e.g. renal cell carcinoma), little is known about the role of this variant in specifying risk for other cancers. In this study, we perform a systematic review and meta-analysis of the published data as a backdrop to a whole-exome sequence(WES)-based characterization of MITF(E318K) risk for various cancers in sporadic samples from the TCGA and several genetically-enriched patient cohorts. We found minimal evidence of MITF(E318K)’s contribution to non-melanoma cancer risk among individuals with low inherited risks of melanoma (OR 1.168; 95% CI 0.78–1.74; p = 0.454), suggesting that earlier reports of an association between this variant and other malignancies may be related to shared environmental or polygenic risk factors rather than MITF(E318K). Interestingly, an association was observed with uterine carcinosarcoma, (OR 9.24; 95% CI 2.08–37.17; p = 0.024), which was not previously described. While more research needs to be completed, this study will help update cancer screening recommendations for patients with the MITF(E318K) variant.

## Introduction

The microphthalmia-associated transcription factor (MITF) is a known master regulator of melanocyte development with functions ranging from pigment production to differentiation, survival, and cell-cycle production of melanocytes. A rare functional variant of MITF(E318K) has been shown to disrupt a conserved SUMOylation site and to confer a two- to fourfold risk for cutaneous melanoma^1,2^. Moreover, there have been small studies suggesting that this variant is also associated with other cancers such as renal cell carcinoma (RCC)^1–13^. However, since epidemiologic observations have documented an association between cutaneous melanoma and many other malignancies, including RCC, it is possible that MITF(E318K) represents a germline “passenger” mutation in these other cancers without directly impacting the risk of these other cancers such as RCC. In this study we perform a systematic review and meta-analysis of the published data as a backdrop to a whole-exome sequence (WES)-based characterization of MITF(E318K) risk for various cancers in sporadic populations from the TCGA database and genetically-enriched patient populations.

## Results

**Figure 1:**
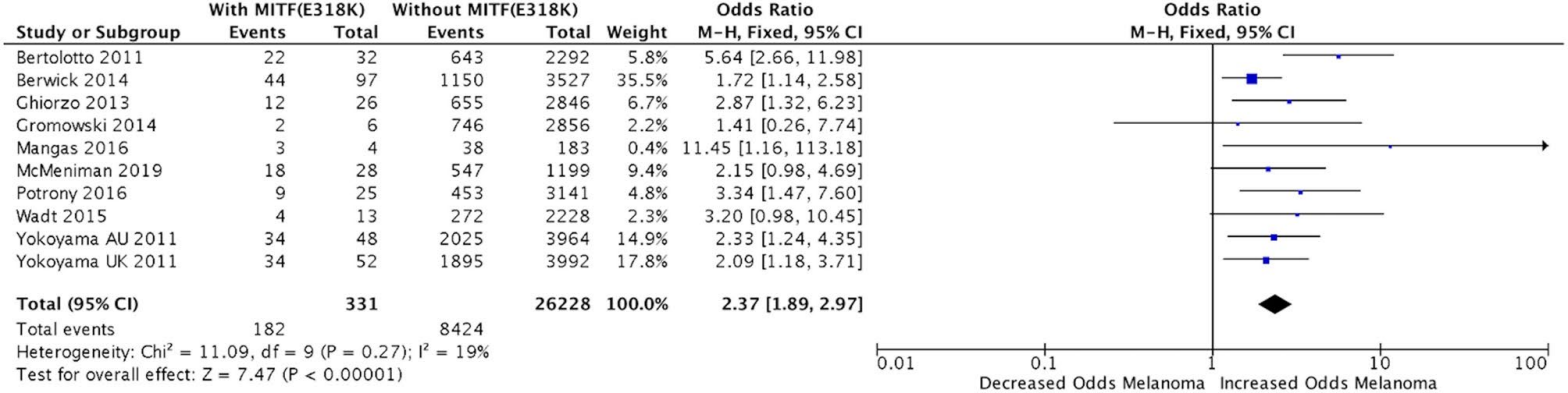
Meta-analysis Odds Ratio of the Association Between MITF(E318K) and Personal History of Melanoma. ^1^Ozola et al. also studied relationship of MITF(E318K) with melanoma, but found no variants in cases or controls and thus was not included in calculations^10^.

Meta-analysis of the data compiled from nine published studies (n = 331)^1–10^ demonstrated that the variant was significantly correlated with melanoma (Fig. 1; odds ratio (OR) 2.37; 95% confidence interval (CI) 1.89–2.97; p < 1E−5; I^2=^19%). Studies were conducted on a mixture of hereditary and sporadic melanoma populations. A few studies identified a relationship of this variant with renal cell carcinoma (RCC), pancreatic cancer, and pheochromocytoma/paraganglioma^2,4,10^. Bertolotto et al. identified over a fivefold increased risk for carriers to develop RCC, melanoma, or both cancers in 829 patients (OR 5.55; 95% CI 2.59–12.91; p = 1.2E−6) and identified a fivefold risk of developing RCC only in 164 “genetically-enriched” patients who were wild-type for RCC-predisposing genes (OR 5.19; 95% CI 1.37–16.87; p = 0.008)^2^. In contrast, other studies did not find an association with sporadic RCC^5,12,13^. Ghiorzo et al. identified a 31-fold increased risk of melanoma in carriers with a personal or family history of pancreatic cancer (OR 30.85; 95% CI 6.85–138.9; p = 0.0005), but did not find significant enrichment of the variant in sporadic pancreatic cancer patients (0/210 patients had the variant)^4^. Castro-Vega et al. screened 555 unrelated pheochromocytoma/paraganglioma patients, and found that the variant was enriched compared to controls (OR 3.19; 95% CI 1.34–7.59; p = 0.005)^11^.

To better understand the risks of this rare functional polymorphism on other cancers, we then systematically evaluated prevalence of the MITF(E318K) variant in multiple cancer cohorts using germline whole-exome sequence data from the TCGA panel and from several genetically-enriched cohorts to validate these associations (Table 1). The TCGA cohort consists of patients mostly with late-onset cancer and unascertained family history, and therefore their risk of comorbid cancers is theoretically low. This set up enables evaluation of primary cancer risk conferred by MITF(E318K). Since the variant is mainly found in the European population (minor allele frequency (MAF) in gnomAD^14^ = 0.00245 for European; MAF = 6.01E−04 for African; MAF = 0 for Asian), we performed the analysis using only European patients identified through principal component analysis (PCA). We used a set of common (MAF > 0.01) LD-pruned variants to perform PCA and used k-means to identify European cluster of samples. The final dataset contained European 6143 cancer cases and 11,106 European non-cancer controls. Comparison of MITF(E318K) frequency in the control sample (MAF = 0.28%, Table 1) matched well with the frequency expectation for Europeans (MAF in gnomAD Europeans = 0.25%), suggesting that our control cohort provides a reference point for MAF free from ancestral bias.

**Table 1:** Relationship of MITF(E318K) with different cancer types in TCGA and genetically-enriched patient cohorts.

Cohort	# MITF(E318K) carriers	N total samples	MAF	Fisher OR (95% CI)	p-value
Control	63	11,106	0.00284	Ref	Ref
TCGA thyroid carcinoma	1	363	0.00138	0.48 (0.05–2.57)	0.724
TCGA glioblastoma	1	326	0.00153	0.54 (0.05–2.86)	1.000
TCGA breast carcinoma	3	776	0.00193	0.68 (0.22–1.96)	0.800
TCGA lung adenocarcinoma	2	501	0.00200	0.70 (0.17–2.63)	1.000
TCGA low grade glioma	2	447	0.00224	0.79 (0.19–2.96)	1.000
TCGA bladder carcinoma	1	209	0.00239	0.84 (0.08–4.50)	1.000
TCGA lung SCC	2	391	0.00256	0.90 (0.22–3.39)	1.000
TCGA/enriched breast cancer	6	976	0.00307	1.08 (0.50–2.39)	0.823
TCGA prostate adenocarcinoma	1	226	0.00221	1.13(0.11–6.55)	0.597
TCGA pheochromocytoma/paraganglioma	1	145	0.00345	1.22 (0.12–6.55)	0.565
TCGA liver carcinoma	1	125	0.00400	1.41 (0.14–7.64)	0.512
TCGA kidney/papillary clear cell carcinoma	1	123	0.00407	1.44 (0.14–7.77)	0.507
TCGA cervical SCC/endocervical adenocarcinoma	1	114	0.00439	1.51 (0.14–8.24)	0.496
TCGA pancreatic adenocarcinoma	1	116	0.00431	1.52 (0.15–8.26)	0.487
TCGA rectum adenocarcinoma	1	113	0.00442	1.57 (0.15–8.49)	0.478
TCGA head/neck SCC	4	436	0.00459	1.62 (0.63–4.23)	0.322
TCGA sarcoma	1	102	0.00490	1.74 (0.17–9.44)	0.444
TCGA colon adenocarcinoma	3	300	0.00500	1.77 (0.58–5.15)	0.251
TCGA uterine corpus endometrial carcinoma	4	385	0.00519	1.79 (0.66–4.83)	0.295
MGH genetically-enriched cutaneous melanoma (GECM)	3	250	0.00600	2.13 (0.69–6.22)	0.178
TCGA cutaneous melanoma	5	409	0.00611	2.17 (0.93–5.29)	0.094
*TCGA cutaneous melanoma* + *MGH GECM*	*8*	*659*	*0.00607*	*2.15 (1.03–4.37)*	*0.061*
MGH genetically-enriched breast cancer	3	200	0.00750	2.67 (0.87–7.83)	0.111
TCGA ocular melanoma	1	47	0.01064	3.81 (0.37–21.69)	0.237
*TCGA uterine carcinosarcoma*	*2*	*39*	*0.02564*	*9.24 (2.08–37.17)*	*0.024*

^1^Cancers whose lower boundary of 95% CI is greater than 1.0 are highlighted in Italics.

The risk of developing any cancer in aggregate with the MITF(E318K) mutation was slightly increased, but this did not reach significance (OR 1.294; 95% CI 0.88–1.88; p = 0.19). Similarly, the risk for all non-melanoma cancers was also increased but not significantly (OR 1.168; 95% CI 0.78–1.74; p = 0.454). Among the 25 cancers tested, uterine carcinosarcoma (OR 9.24; 95% CI 2.08–37.17; p = 0.024) and melanoma (OR 2.15; 95% CI 1.03–4.37; p = 0.061) exhibited the strongest associations with the variant. We did not find any significant association of MITF(E318K) with renal cell carcinoma (RCC) (p = 0.5068), pancreatic cancer (p = 0.487), or pheochromocytoma/paraganglioma (p = 0.565) in the TCGA cohort.

Further, we tested whether differences in ascertainment between cancer cohorts could explain the lack of the previously reported association with RCC. Specifically, it is expected that the burden of germline risk variants should be lower in cohorts with unascertained family history (TCGA) than in genetically-enriched samples. We compared allele frequencies of MITF (E318K) for the familial (MAF = 0.6%) and sporadic (MAF = 0.6%) melanoma cohorts and did not observe any difference, suggesting that the sporadic cohort should be powerful enough to detect primary cancer risks associated with MITF(E318K).

Therefore, in our analysis of RCC, pancreatic cancer, pheochromocytoma/paraganglioma cohorts, there does not appear to be a strong risk of primary cancer conferred by MITF(E318K). Importantly, TCGA non-melanoma cohorts are unlikely to have substantial inherited risk of cutaneous melanoma due to the relatively high age of participants and no ascertainment, resulting in the lack of an association signal for MITF(E318K). Previous studies reporting the non-melanoma cancer association of MITF(E318K) have not yet assessed inherited melanoma risks for the RCC and other cancer cohorts.

## Discussion

This study is an important addition to the published literature as it represents the largest MITF(E318K) variant analysis to date with more samples collectively between cases and controls than found in previously published studies. In addition, with WES, we utilize principal component analysis (PCA) to match for European ancestry which allows for a much cleaner calculation of risk compared to most other studies that relied solely on MITF(E318K) counts without regard for population mix. Although we corroborated the association of the variant with melanoma, we found minimal evidence of MITF(E318K)’s contribution to the risk of non-melanoma cancers among individuals with low inherited risks of melanoma.

These results suggest that earlier reports of an association between this variant and other malignancies may be related to shared environmental or polygenic risk factors rather than this specific MITF polymorphism. For example, multiple studies have shown an association between RCC and melanoma. In a 2018 analysis of the Surveillance, Epidemiology, and End Results (SEER) database, the Standardized Incidence Ratio (SIR) for developing secondary primary melanoma among RCC patients was 2.31, and the SIR for developing secondary primary RCC among melanoma patients was 2.87^15^. In previous MITF(E318K) studies, inclusion of high melanoma risk patients with a primary manifestation of RCC or other cancers could have driven increased frequency of MITF(E318K) in observed non-melanoma cohorts. Common putative risk genes for RCC and melanoma – *BAP1, MITF, CDKN2B* – suggest that similar pathways are disrupted in both disorders; however, individual variant risks should be evaluated when shared genetic background is taken into account. For example, Christensen et al*.* investigated 48 families with early onset RCC, a family history of RCC, a family history of RCC and melanoma, or both RCC and melanoma diagnosis in the same individual. MITF(E318K) was found only in a RCC-affected member of a family with multiple melanomas. Consequently, authors concluded that *BAP1*, *MITF* or *CDKN2B* are not frequent causes of hereditary renal cancer^12^.

This common polygenic background and shared environmental factors likely contributed, at least in part, to Bertolotto et al*.*’s observation of an enrichment of the variant in RCC and melanoma cases^2^. While this study did report an association with RCC-only, the cohort was young, had family history of RCC, and had rare histological subtypes, unlike the TCGA patients. Thus, MITF(E318K) may play a role in driving cancer formation in a subset of “genetically-enriched” RCC patients by a still undisclosed biological mechanism. Of note, several other researchers have also failed to identify an association of sporadic RCC with MITF(E318K)^5,12,13^. This may also explain Ghiorzo et al.’s findings, as their association was only noted in melanoma patients with a personal or family of pancreatic cancer, and not in patients with sporadic pancreatic cancer. The underpinnings of variance regarding pheochromocytoma/paraganglioma is unclear, but may be due to the difference in age between our and Castro-Vega et al.’s cohorts.

Beyond cutaneous melanoma, our study also found a putative risk association between the germline MITF(E318K) and uterine carcinosarcoma (malignant mixed Müllerian tumors) although the low frequency of the variant and, expectedly, low numbers of variant carriers, preclude us from rigorously adjusting for multiple hypotheses. Interestingly, uterine carcinosarcomas (mixed Mullerian tumors) have been reported to exhibit melanocytic differentiation, and increased MITF expression^16,17^. While more research needs to be completed, this study will help update cancer screening recommendations for patients with the MITF(E318K) variant.

A limitation of this study is the relatively small sample size of some of the TCGA cohorts. This, along with the rarity of the MITF variant, contributes to the limited power of analysis. Nevertheless, it may be worthwhile to better characterize the relationship of MITF(E318K) with different cancer types in a larger study with explicit evaluation of inherited polygenic predisposition to melanoma, and to look for biology of how MITF(E318K) mediates risks for co-occurrence of other cancers with melanoma.

## Methods

### Literature review and meta-analysis

We searched MEDLINE and Harvard HOLLIS from database inception to February 25, 2020 by using various combinations of the terms “melanoma”, “MITF”, “E318K”, and “cancer”. Studies without non-cancer controls were excluded. No pertinent non-English articles were found. Meta-analysis was conducted using the Review Manager 5.3 software, published by the Cochrane community. All variables were considered dichotomous, and analyzed using the Mantel–Haenszel statistical method and fixed effect analytic method. Heterogeneity was assessed using the Chi^2^ test and I^2^ statistic. See Supplementary File S1 for full methods and excluded studies.

### TCGA and enriched cohorts

Datasets of germline cancer (TCGA sporadic and MGH genetically-enriched cases) and control exome sequences are described in previous publications from our lab^18,19^. Details of enriched patient cohorts can be found in Supplementary Methods S1. All datasets can be accessed through dbGAP using the following accession numbers: phs000178.v1.p1, phs000823.v1, phs000822.v1.p1, phs000806.v1.p1, and phs000814.v1.p1.

Aggregated set of samples was used for joint variant calling using Picard/GATK pipeline. Principal component analysis (PCA) using common LD-pruned autosomal variants was used to identify a cluster of European samples (k-means). Individual genotypes for the MITF(E318K) variant were filtered. Only genotypes with depth of coverage more than 10X and genotype quality more than 20 × were used for analysis and all cohorts had the target variant called in more than 95% of the samples. Fisher exact test (two-sided) was used to test association with a specific cancer type. Odds ratio and 95% confidence interval was calculated using Graph Prism using the Baptista-Pike method. MAF was calculated by MITF(E318K) # Minor Alleles/# Total Samples/2.

## Supplementary information

Supplementary file 1

## Data Availability

The TCGA datasets were generated using the data publically available at https://portal.gdc.cancer.gov. The genetically-enriched datasets can be accessed through dbGAP using the following accession numbers: phs000178.v1.p1, phs000823.v1, phs000822.v1.p1, phs000806.v1.p1, and phs000814.v1.p1.
