# Supplementary material for: Cancer risks associated with the germline MITF(E318K) variant
Source: Sci Rep. 2020 Oct 13;10:17051. doi: 10.1038/s41598-020-74237-z (PMC7555480; doi:10.1038/s41598-020-74237-z)
Supplement: Supplementary file 1 [file 41598_2020_74237_MOESM1_ESM.docx]

# **Cancer Risks Associated with the Germline MITF(E318K) Variant**

Samantha M. Guhan BA^1^, Mykyta Artomov PhD^2^, Shelley McCormick MS LCGC^3^, Ching-Ni Njauw MS^1^, Alexander J. Stratigos MD^4^, Kristen Shannon MS LCGC^3^, Leif W. Ellisen MD PhD^3^, Hensin Tsao MD PhD^1,3*^

^1^Wellman Center for Photomedicine at Massachusetts General Hospital, Boston, MA

^2^MGH Analytic and Translational Genetics Unit, MGH and Broad Institute, Boston, MA

^3^Massachusetts General Hospital Cancer Center, Boston, MA

^4^First Department of Dermatology-Venereology, National and Kapodistrian University of Athens, Faculty of Medicine, 'A. Sygros' Hospital for Cutaneous & Venereal Diseases, Athens, Greece

**Supplementary File:**

**Systematic Review and Meta-Analysis Additional Information**

*Reporting of Methods*

All included studies for qualitative review were case-control studies. A study was deemed relevant if it studied the frequency of the MITF(E318K) mutation in a melanoma and control population. Cases and controls needed to be taken from comparable populations to minimize confounders. Any study that did not include a reference group of non-cancer controls was excluded. Case reports were excluded. No pertinent non-English articles were found. Reference lists of obtained articles were studied.

Given the small number of studies found, there is a high risk for publication bias.

Table 1: Excluded Studies from Melanoma Quantitative Meta-Analysis and Reason Excluded

| Study | Reason Excluded |
| --- | --- |
| Bassoli et al 2018^1^ | Case report of a single patient |
| Bruno et al 2016^2^ | Did not include a reference group of non-cancer controls; instead compared prevalence of MITF(E318K) mutation in multiple primary melanomas vs. single primary melanoma |
| Christensen et al 2019^3^ | Did meet inclusion criteria for qualitative synthesis of relationship with RCC but not for melanoma; studied individuals with early onset RCC, a family history of RCC, a family history of RCC and melanoma, or both RCC- and melanoma diagnosis in the same individual |
| Ciccarese et al 2020^4^ | Did not include a reference group of non-cancer controls; instead compared MITF mutant melanomas with MITF WT melanomas |
| Duffy et al 2020^5^ | Did not list number of controls from the UK Biobank who tested positive for the MITF(E318K) mutation |
| Muller et al 2016^6^ | Did not include a reference group of non-cancer controls; only screened 123 high-risk melanoma patients for the variant |
| Ozola et al 2015^7^ | Did meet inclusion criteria for qualitative synthesis; however, found no variants in cases and controls and thus was excluded from calculations |
| Pollio et al 2014^8^ | Only studied variant status in 12 patients with multiple primary melanomas of the head and neck |
| Potjer et al 2019^9^ | Did not include a reference group of non-cancer controls for this variant (only included sequencing data for MC1R in non-cancer controls) |
| Sturm et al 2014^10^ | Did not include a reference group of non-cancer controls; instead compared MITF mutant melanomas with MITF WT melanomas |

**Enriched Patient Cohorts Additional Information**

Datasets of germline cancer and control exome sequences are described in previous publications from our lab (PMID: 30718883 and PMID: 29522175). All datasets can be accessed through dbGAP using the following accession numbers: phs000178.v1.p1, phs000823.v1, phs000822.v1.p1, phs000806.v1.p1, and phs000814.v1.p1.

The MGH genetically enriched cutaneous melanoma (GECM) cohort consisted of two groups of samples:

(1) patients who were seen at the MGH and who had a histologically-proven CM AND

a. at least one 1st degree affected relative

OR

b. >2 affected relatives on one side of the family regardless of degree of relationship (proband CM + relative with CM, “Familial CM/CM”; proband CM + relative with OM, “Familial CM/OM”)

OR

c. >3 primary melanomas regardless of family history (“MPM CM-CM”).

(2) patients evaluated at the A. Sygros Hospital in Athens, Greece and who had a histologically-proven CM AND

a. >1 affected relative on one side of the family (“Familial CM/CM”)

OR

b. >2 primary melanoma (“MPM CM-CM”).

The MGH genetically enriched breast cancer cohort consisted of patients who had a histologically-proven breast cancer AND

a. at least one 1st degree affected relative

OR

b. age at clinical diagnosis less than 40 years old

References

1 Bassoli, S. *et al.* Clinical, dermoscopic, and confocal features of nevi and melanomas in a multiple primary melanoma patient with the MITF p.E318K homozygous mutation. *Melanoma Res* **28**, 166-169, doi:10.1097/CMR.0000000000000427 (2018).

2 Bruno, W. *et al.* Multiple primary melanomas (MPMs) and criteria for genetic assessment: MultiMEL, a multicenter study of the Italian Melanoma Intergroup. *J Am Acad Dermatol* **74**, 325-332, doi:10.1016/j.jaad.2015.09.053 (2016).

3 Christensen, M. B. *et al.* Exploring the hereditary background of renal cancer in Denmark. *PLoS One* **14**, e0215725, doi:10.1371/journal.pone.0215725 (2019).

4 Ciccarese, G. *et al.* Clinical, pathological and dermoscopic phenotype of MITF p.E318K carrier cutaneous melanoma patients. *J Transl Med* **18**, 78, doi:10.1186/s12967-020-02253-8 (2020).

5 Duffy, D. L. *et al.* Genes Determining Nevus Count and Dermoscopic Appearance in Australian Melanoma Cases and Controls. *J Invest Dermatol* **140**, 498-501 e417, doi:10.1016/j.jid.2019.05.032 (2020).

6 Muller, C. *et al.* Characterization of patients at high risk of melanoma in Austria. *Br J Dermatol* **174**, 1308-1317, doi:10.1111/bjd.14407 (2016).

7 Ozola, A. & Pjanova, D. The lack of E318K MITF germline mutation in Latvian melanoma patients. *Cancer Genet* **208**, 355-356, doi:10.1016/j.cancergen.2015.03.011 (2015).

8 Pollio, A. *et al.* Multiple primary melanomas versus single melanoma of the head and neck: a comparison of genetic, diagnostic, and therapeutic implications. *Melanoma Res* **24**, 267-272, doi:10.1097/CMR.0000000000000057 (2014).

9 Potjer, T. P. *et al.* Multigene panel sequencing of established and candidate melanoma susceptibility genes in a large cohort of Dutch non-CDKN2A/CDK4 melanoma families. *Int J Cancer* **144**, 2453-2464, doi:10.1002/ijc.31984 (2019).

10 Sturm, R. A. *et al.* Phenotypic characterization of nevus and tumor patterns in MITF E318K mutation carrier melanoma patients. *J Invest Dermatol* **134**, 141-149, doi:10.1038/jid.2013.272 (2014).
